# Association between nutritional targets and muscle index changes in patients with terminal cancer under palliative care

**DOI:** 10.55730/1300-0144.6074

**Published:** 2025-09-11

**Authors:** İrem KIRAÇ UTKU, Burcu ÇALIŞKAN AYDOĞMUŞ, Hatice Nur AVCIOĞLU, Müge ÇATIKKAŞ, Ayfer DURAK, Deniz SEVİNDİK GÜNAY, Umut SAFER

**Affiliations:** 1 Division of Internal Medicine, Department of Internal Medicine, Tekirdağ İsmail Fehmi Cumalıoğlu City Hospital, Tekirdağ, Turkiye; 2 Division of Geriatrics, Department of Internal Medicine, Tekirdağ İsmail Fehmi Cumalıoğlu City Hospital, Tekirdağ, Turkiye; 3 Department of Nutrition and Dietetics, University of Health Sciences, Sancaktepe Şehit Prof. Dr. İlhan Varank Training and Research Hospital, İstanbul, Turkiye; 4 Department of Nutrition and Dietetics, University of Health Sciences, Sancaktepe Şehit Prof. Dr. İlhan Varank Training and Research Hospital, İstanbul, Turkiye; 5 Division of Geriatrics, Department of Internal Medicine, University of Health Sciences, Sancaktepe Şehit Prof. Dr. İlhan Varank Training and Research Hospital, İstanbul, Turkiye; 6 Division of Geriatrics, Department of Internal Medicine, Sabuncuoglu Serefeddin Varank Training and Research Hospital, Amasya, Turkiye; 7 Division of Geriatrics, Department of Internal Medicine, University of Health Sciences, Sancaktepe Şehit Prof. Dr. İlhan Varank Training and Research Hospital, İstanbul, Turkiye; 8 Division of Geriatrics, Department of Internal Medicine, University of Health Sciences, Sancaktepe Şehit Prof. Dr. İlhan Varank Training and Research Hospital, İstanbul, Turkiye

**Keywords:** Quadriceps muscle, rectus femoris, terminal care, malnutrition

## Abstract

**Background/aim:**

Malnutrition and muscle wasting are common in terminal cancer patients and adversely affect prognosis. Bedside muscle ultrasonography (US) has emerged as a practical tool to assess nutritional status and monitor therapy. This study evaluated the relationship between achieving individualized nutritional targets and changes in muscle indices measured by US in terminal cancer patients receiving palliative care.

**Materials and methods:**

Subcutaneous fat tissue thickness (SFTT), muscle thickness (MT), and cross-sectional area (CSA) of the rectus femoris (RF) and biceps brachii (BB) muscles were assessed by US over 10 days and normalized to height squared (m^2^). A total of 95 patients with complete retrospective data were included; 74.7% achieved their caloric targets, while 25.3% did not.

**Results:**

Patients who failed to meet caloric goals exhibited significantly greater reductions across all muscle parameters. Among those who achieved caloric targets, protein intake ≥1.5 g/kg/day was associated with reduced muscle loss compared to lower intake. The RF muscle was particularly sensitive to nutritional status, exhibiting more pronounced changes in response to both caloric and protein adequacy. No significant differences were observed between feeding routes, although patients receiving parenteral nutrition tended to exhibit greater decline. Correlation analyses demonstrated a significant negative association between time to achieve caloric targets and muscle preservation, underscoring the importance of early intervention. Linear regression further identified delayed caloric achievement as an independent predictor of accelerated muscle loss.

**Conclusion:**

Achieving caloric and high-protein targets is critical for preserving muscle mass in terminal cancer patients. US, particularly of the RF muscle, provides a reliable, noninvasive, and practical tool for monitoring the effects of nutritional interventions in this vulnerable population.

## 1. Introduction

Patients in the terminal stage of cancer are affected not only by the progressive impact of the malignancy but also by systemic deterioration caused by treatment. During this period, the prevalence of malnutrition, sarcopenia, and catabolic processes significantly limits patients’ physical functions and substantially reduces their quality of life. Malnutrition and sarcopenia, particularly in patients with advanced-stage cancer, are strongly associated with higher complication rates, lower performance status, prolonged hospitalization, and increased mortality [1,2]. Among patients receiving care in palliative units during the terminal phase, nutritional status is crucial both for predicting prognosis and for prolonging survival [3]. In this context, muscle tissue directly reflects an individual’s nutritional status and can change rapidly, particularly in response to protein intake. A decrease in muscle mass has been associated with reduced functional capacity, increased risk of complications, and poorer clinical outcomes [4]. Thus, muscle assessment has gained importance in recent years for monitoring the effectiveness of nutritional therapy.

Although imaging techniques such as computed tomography (CT), magnetic resonance imaging (MRI), and dual-energy X-ray absorptiometry (DEXA) are considered gold-standard methods for evaluating muscle mass, they are costly, have limited accessibility, and can be difficult to use for patient transport in certain situations [5]. Therefore, muscle US has recently emerged as a noninvasive, portable, repeatable, and radiation-free method that can be performed at the bedside. Given the need for bedside assessments without moving patients in palliative care settings, muscle US has become highly valuable in clinical practice. Ultrasonographic measurements enable the direct and reliable evaluation of parameters such as MT, CSA, and SFTT [6,7]. Previous studies have shown that this method is valid and reliable for assessing muscle mass and function [8,9]. Ultrasonographic muscle measurements are also used to evaluate the effectiveness of nutritional therapy, and changes in muscle mass are accepted as objective indicators of treatment response. To minimize the impact of individual differences in body size, it has been recommended to normalize muscle parameters to height squared [10].

Achieving caloric and protein targets is crucial for evaluating the effectiveness of nutritional therapy [11]. According to the guidelines of the European Society for Clinical Nutrition and Metabolism (ESPEN), the recommended daily energy intake for cancer patients is 25–30 kcal/kg, with a minimum protein requirement of 1 g/kg/day and an optimal target of ≥1.5 g/kg/day [12].

This study aims to examine the relationship between the achievement of individualized energy and protein goals and muscle US findings in patients with terminal-stage malignancies receiving care in palliative units. The effects of caloric and protein intake on muscle mass were evaluated over a 10-day period by assessing changes in SFTT, MT, and CSA parameters measured in the RF and BB muscles. Additionally, the roles of different feeding routes (oral nutrition (OR), enteral nutrition (EN), parenteral nutrition (PN), combined) and the time required to achieve caloric goals in relation to muscle mass were analyzed.

Literature reports indicate that muscle mass loss in critically ill and terminally ill patients can be detected using ultrasound even with short-term monitoring. A metaanalysis of 52 studies in intensive care populations showed that patients lost 1.75% (95% CI: −2.05 to −1.45) of RF-MT or 2.10% (95% CI: −3.17 to −1.02) per day [13]. These data suggest that significant muscle loss can also occur rapidly in terminal patients receiving palliative care and that such changes can be monitored noninvasively using US.

The primary objective of this study is to elucidate the relationship between routine nutritional assessments and muscle US findings in patients with terminal-stage cancer. Thus, this study aims to provide scientific evidence for the role of nutritional monitoring in preserving muscle integrity in clinical practice. The hypothesis of this study is that adequate energy and protein intake reduces muscle loss.

## 2. Materials and methods

### 2.1. Ethical approval and study design

This study was approved by the Clinical Research Ethics Committee of the University of Health Sciences, Şehit Prof. Dr. İlhan Varank Training and Research Hospital, on 8 May 2024 (decision no: 2024/137). The study was conducted as a single-center, retrospective, observational study.

### 2.2. Patient selection and enrollment process

A total of 1175 patient records from the palliative care unit were screened. Of these, 572 patients were diagnosed with terminal-stage malignancy. However, 477 patients were excluded for the following reasons:

- Early death before completion of the 10-day follow-up (n = 21)
- Early discharge from the palliative care unit before the second US assessment (n = 102)
- Delayed second US **(>**10 days) due to nonmedical reasons such as public holidays, temporary leave permits, or other medical procedures that postponed the assessment (n = 203)
- Additionally, 151 patients were excluded due to incomplete nutritional or ultrasonographic records, most commonly because of missing daily caloric/protein intake documentation or absence of baseline US within the first 3 days of admission.

The final study population consisted of 95 patients who met all inclusion criteria and had complete paired ultrasonographic measurements at baseline and on day 10. To enhance transparency, the patient selection process is presented in a flowchart (Figure).

A total of 95 patients with complete datasets were included. The following patient data were collected and assessed:

- Nutritional Risk Screening 2002 (NRS-2002) score ≥3, indicating risk of malnutrition and initiation of nutritional support by dietitians.
- Age, sex, height, weight, and Eastern Cooperative Oncology Group (ECOG) performance scale score.
- Maximum net daily caloric and protein intake attainable within 10 day of admission.
- Feeding route (oral, enteral, parenteral, or combined).
- Ultrasonographic measurements of the RF and BB muscles performed within the first 3 days of hospitalization and repeated on day 10.

### 2.3. Inclusion criteria

Patients diagnosed with metastatic terminal malignancy who were deemed unsuitable for systemic treatment or whose treatment was discontinued by an oncology specialist.

Patients who received nutritional support initiated by dietitians.

Patients with complete records of daily net caloric and protein intake, feeding route, NRS-2002 score, demographic data (age, sex, height, weight), and serial muscle US measurements.

### 2.4. Exclusion criteria

Patients with incomplete data as described above.

Patients without a diagnosis of terminal-stage malignancy.

Patients receiving systemic anticancer therapy.

Patients whose nutritional support was interrupted for clinical reasons.

### 2.5. Nutritional assessment

Individual caloric and protein requirements for each patient were determined on the day of admission by two experienced clinical dietitians, with strict adherence to ESPEN guidelines for cancer patients. Daily energy needs were calculated as 25–30 kcal/kg/day, and protein requirements were set at a minimum of 1.0 g/kg/day, with an optimal target of ≥1.5 g/kg/day. Actual body weight was used for calculations in most patients; however, in obese patients (BMI ≥ 30 kg/m^2^), adjusted body weight was calculated using the following formula:


Adjusted body weight (kg)=Ideal body weight+0.25×(Actual body weight-Ideal body weight)

A standardized institutional protocol was followed to initiate and advance nutritional support. Nutritional therapy generally commenced at approximately 50% of the calculated target on the first day and was gradually increased to 100% of the requirement over the following 48–72 h, depending on patient tolerance and metabolic stability. Nutritional support was delivered via oral, enteral, parenteral, or combined routes, depending on clinical indication and patient preference.

A team of two dedicated dietitians visited each patient daily to monitor tolerance, intake, and any interruptions to feeding. All daily net caloric and protein intakes were documented in a structured nutritional monitoring chart that included the feeding route, type of formula or dietary plan, and any modifications made during the day. The chart also documented reasons for reduced intake (e.g., gastrointestinal intolerance, clinical deterioration, or feeding access problems) and was used to calculate the percentage of the target achieved for both energy and protein.

Adherence to nutritional targets was assessed daily. For each patient, the number of days required to reach full caloric intake was recorded. These systematically collected daily records formed the basis for the retrospective evaluation of nutritional adequacy and its association with changes in muscle mass.

Body weight (kg) and height (m) of included patients were obtained from medical records, and body mass index (BMI) was calculated for each individual using the following formula:

-
$\text{BMI }(\text{kg}/{\text{m}}^{2})=\text{Body weight }(\text{kg})/{\text{Height}}^{2}\;({\text{m}}^{2})$

### 2.6. Muscle ultrasonography assessment

According to our clinical protocol, muscle US was performed on all patients by a single experienced geriatrician within the first 3 days of hospitalization and every 10 days thereafter, provided the patient remained stable. These US measurements were performed at the bedside within the hospital for scientific purposes. The ultrasonographic evaluation followed the recommendations of the European Union Geriatric Medicine Society Sarcopenia Special Interest Group. Measurements were performed using the Philips Affiniti 50 Ultrasound system (Philips Healthcare, Amsterdam, Netherlands) with a 5 cm wide, 7.5 MHz linear probe in B-mode. While patients lay supine with limbs extended and relaxed, the SFTT, MT, and CSA of the RF and BB muscles were measured. After a 5 min rest period, each measurement was repeated three times, and the average value was recorded as the final measurement. The RF muscle was evaluated at the distal one-third point between the anterior inferior iliac spine and the proximal patellar line, while the BB muscle was measured at the midpoint between the acromion and olecranon. The US probe was placed perpendicular to the transverse axis of the thigh and humerus, applying minimal pressure. The distance between the skin and superficial fascia of the RF and BB muscles was defined as SFTT in millimeters. The distance between the superficial and deep fascia of the muscles was defined as MT. The cross-sectional area (CSA) between the superficial and deep fascia of the muscles was recorded in cm^2^.

Muscle parameters assessed by US:

- Subcutaneous fat tissue thickness of the rectus femoris (RF-SFTT)
- Muscle thickness of the rectus femoris (RF-MT)
- Cross-sectional area of the rectus femoris (RF-CSA)
- Subcutaneous fat tissue thickness of the biceps brachii (BB-SFTT)
- Muscle thickness of the biceps brachii (BB-MT)
- Cross-sectional area of the biceps brachii (BB-CSA)

All patients had their initial and follow-up US data recorded at admission and on day 10, respectively. Ultrasonographic measurements of SFTT, MT, and CSA obtained for muscle mass evaluation were normalized to account for interindividual differences in body size. To achieve normalization, all muscle measurements were divided by the square of the patient’s height in meters (height^2^), yielding index values. US evaluations were consistently performed by the same physician.

This method is widely accepted in the literature for muscle mass evaluations normalized to body surface area.

Calculated muscle index formulas:

-
$\text{Cross-sectional area index }(\text{CSA-I})\;({\text{cm}}^{2}/{\text{m}}^{2})=\text{CSA }({\text{cm}}^{2})/{\text{Height}}^{2}\;({\text{m}}^{2})$
-
$\text{Muscle thickness index }(\text{MT-I})\;(\text{cm}/{\text{m}}^{2})=\text{MT }(\text{cm})/{\text{Height}}^{2}\;({\text{m}}^{2})$
-
$\text{Subcutaneous fat tissue thickness index }(\text{SFTT-I})\;(\text{cm}/{\text{m}}^{2})=\text{SFTT }(\text{cm})/{\text{Height}}^{2}\;({\text{m}}^{2})$

These indices facilitated objective and standardized comparisons between patients.

### 2.7. Data analysis

Patients were categorized into two groups based on whether they achieved caloric goals. Additionally, patients who achieved caloric targets were subdivided into two groups based on protein intake levels (1.0–1.49 g/kg/day and ≥1.5 g/kg/day).

Muscle loss (differences in US measurements), energy and protein intake, and nutritional methods were compared between groups. The collected data were evaluated using statistical analyses.

### 2.8. Statistical analysis

In the statistical analysis, demographic data (age, sex, cancer type), body mass index (BMI), NRS-2002 scores, time to achieve maximum caloric goals, and feeding routes (oral, enteral, parenteral, or combined) were evaluated. Descriptive statistics were also presented for the SFTT, MT, and CSA values obtained from the baseline measurements of the RF and BB muscles.

The differences between the first and second measurements were calculated, and change scores were generated for each parameter. Skewness and kurtosis values of these difference variables were examined, and a threshold of ±3 was used to assess conformity to the normal distribution [14]. In evaluating normality, graphical methods such as histograms and boxplots were used only for supportive purposes, whereas numerical criteria (skewness and kurtosis coefficients) were primarily considered. Furthermore, the homogeneity of variances, a prerequisite for the parametric tests used in group comparisons, was assessed using Levene’s test.

For comparisons between two groups, the independent samples t-test was used when normality and homogeneity of variance assumptions were met; otherwise, the Mann–Whitney U test was applied. For three or more groups, one-way ANOVA was conducted when the normality assumption was met, whereas the Kruskal–Wallis H test was used when the assumption was not satisfied. Post hoc Bonferroni-adjusted comparisons were performed following significant ANOVA results, and Dunn–Bonferroni pairwise comparisons were conducted after significant Kruskal–Wallis tests.

For associations between quantitative variables, Pearson’s correlation coefficient (r) was used when the normality assumption was satisfied, whereas Spearman’s rank correlation coefficient (ρ) was applied otherwise. To assess potential predictors of differences in RF-CSA-I, a multivariable linear regression model including sex, age, NRS-2002 score, feeding route, and ECOG performance status was performed.

All analytical results were reported as mean (M), standard deviation (SD), median (Med), quartile values (Q1–Q3), minimum (Min), and maximum (Max) for quantitative data, and as frequency (n) and percentage (%) for categorical data. The statistical significance level was set at p < 0.05. The type I error rate (α) was set at 5%, and the type II error rate (β) was set at 20% (statistical power = 80%).

Given the retrospective design of this study, a post hoc power analysis was conducted using G*Power 3.1 (Heinrich-Heine-Universitat Düsseldorf, Düsseldorf, Germany) to determine the achieved statistical power. The analysis was based on the observed difference in the rectus femoris cross-sectional area index between caloric intake groups, which was selected as the primary outcome variable for power calculation. With a significance level of α = 0.05, the post hoc power analysis revealed an achieved statistical power of 99.8%

## 3. Results

The mean age of participants was 74.09 years (SD = 10.37); the mean Nutritional Risk Screening-2002 (NRS-2002) score was 5.01 (SD = 0.89); the average time to reach caloric targets was 7.0 days (SD = 2.81); and the mean body mass index (BMI) was 22.39 kg/m^2^ (SD = 5.08). Among the participants, 55.8% were male (n = 53) and 44.2% were female (n = 42). A total of 74.7% of patients (n = 71) achieved their caloric intake targets, whereas 25.3% (n = 24) did not. Regarding feeding routes, 24.21% received parenteral nutrition, 30.52% enteral nutrition, 34.73% oral nutrition, and 10.52% a combination of parenteral and oral nutrition (Table 1).

According to the post hoc power analysis performed with G*Power 3.1 (Heinrich-Heine Universitat Düsseldorf, Düsseldorf, Germany), the achieved statistical power of the study was 99.8%.

The distribution of participants by tumor type showed that lung cancer had the highest proportion (23.1%), followed by central nervous system tumors (12.6%), gastric cancer (10.5%), pancreatic cancer (8.4%), prostate cancer (8.4%), and breast cancer (6.3%). The distribution of ECOG performance status was as follows: zero in 17 patients (17.9%), one in 22 (23.2%), two in 16 (16.8%), three in 12 (12.6%), and four in 28 (29.5%). All muscle parameters showed a similar pattern of reduction in the second measurement (Table 1).

Muscle index differences between participants who achieved their caloric targets (n = 71) and those who did not (n = 24) showed statistically significant differences across all parameters. The BB-CSA-I was −0.272 (SD = 0.145) in the group that failed to meet the caloric target, compared to −0.054 (SD = 0.138) in the group that achieved the target (p < 0.001). Similarly, BB-MT-I values showed significantly greater reductions in the group that did not achieve their caloric target (p = 0.001).

Regarding RF measurements, RF-CSA-I and RF-MT-I were also more markedly reduced in the group that failed to meet their caloric targets (p = 0.001 and p < 0.001, respectively). Furthermore, both RF-SFTT-I and BB-SFTT-I showed significantly greater reductions in the nonachieving group (p = 0.007 and p < 0.001, respectively) (Table 2).

Among the 71 participants who achieved their caloric targets, those with a daily protein intake <1.5 g/kg had a BB-CSA-I difference of −0.087 (SD = 0.137), compared to −0.023 (SD = 0.132) in the group consuming ≥1.5 g/kg/day of protein (p = 0.048). Similarly, BB-MT-I differences were significantly smaller in the high-protein intake group, indicating reduced muscle loss (p = 0.008). RF-CSA-I and RF-MT-I differences were also significantly smaller in the ≥1.5 g/kg/day protein group (both p < 0.001). Specifically, the RF-CSA-I difference was −0.083 (SD = 0.069) in the lower-protein group, compared to −0.001 (SD = 0.073) in the higher-protein group. No statistically significant differences in SFTT-I were observed in either the BB or RF regions according to protein intake level (p = 0.082 and p = 0.317, respectively) (Table 3).

Correlations between daily protein intake and muscle index differences among participants who achieved their caloric target were examined. Among these participants, daily protein intake (g/kg/day) showed significant positive correlations with RF muscle indices. Specifically, a moderate and significant correlation was observed between RF-CSA-I and protein intake (r = 0.507; p < 0.001). Similarly, RF-MT-I was significantly and positively correlated with protein intake (r = 0.485; p < 0.001). The relationship between RF-SFTT-I and protein intake was positive but weak (p = 0.249; p = 0.036). For BB muscle indices (CSA-I and MT-I), positive but nonsignificant correlations with protein intake were found (p > 0.05). In contrast, BB-SFTT-I demonstrated a negative correlation with protein intake (r = −0.139; p = 0.247) (Table 4).

The results of muscle index comparisons according to participants’ nutritional categories are presented below. No statistically significant differences in muscle index values were observed across feeding routes (p > 0.05) (Table 5).

Multivariable linear regression analysis for RF-CSA-I (difference) did not reach statistical significance (F = 1.368; p = 0.215), showing low explanatory power (adjusted R^2^ = 0.034) and a Durbin–Watson coefficient of 1.870, which indicated no autocorrelation. Age, sex, NRS-2002 score, feeding route, and ECOG performance status were included as independent variables; however, none demonstrated a statistically significant association with RF-CSA-I (difference). Among these, the feeding route “PN + OR” showed a trend toward a negative association (B = −0.081, 95% CI: −0.169 to 0.006, p = 0.067), but this did not reach statistical significance. Other variables, including age (p = 0.922), sex (p = 0.459), and NRS-2002 (p = 0.815), were not associated with the outcome (Table 6).

In patients who achieved their caloric targets, analysis of the number of days required to reach maximum intake was revealed that delayed achievement was significantly associated with adverse changes in specific muscle indices. BB-MT-I (ρ = −0.468; p < 0.001) and BB-CSA-I (r = −0.419; p < 0.001) were significantly and inversely correlated with delayed achievement of the caloric target. Significant negative correlations were also observed in RF muscle measurements. RF-MT-I (r = −0.381; p < 0.001) and RF-CSA-I (ρ = −0.273; p = 0.007) were significantly and inversely correlated with the number of days required to achieve the caloric target. A weak, negative, but statistically nonsignificant correlation was observed between RF-SFTT-I and the number of days required to achieve the caloric target (ρ = −0.177; p = 0.086). The correlation between BB-SFTT-I and the number of days required to achieve the caloric target was weakly negative and not statistically significant (r = −0.137; p = 0.185) (Table 7).

## 4. Discussion

This study evaluated the relationship between the achievement of individualized nutritional targets and ultrasonographic measurements of muscle mass in patients with terminal-stage malignancy. The findings highlight the impact of nutritional support on muscle integrity during palliative care. Current literature emphasizes the reliability of US for assessing muscle mass. Tillquist et al. reported that RF-CSA measured by ultrasound in intensive care patients reliably reflected changes in muscle mass and significantly predicted functional status [15]. A systematic review by Casey et al. demonstrated that muscle measurements obtained via US showed strong correlations with reference methods such as CT and MRI [16]. Similarly, in our study, muscle CSA and MT were measured using US and normalized by height squared (height^2^), allowing for comparative analyses. This approach is consistent with the recommendations of the European Working Group on Sarcopenia in Older People (EWGSOP), which advocate evaluating muscle mass normalized by height squared (height^2^) to minimize interindividual variability due to body size and enhance the reliability of comparative analyses [10]. Beyond conventional manual measurements, recent technological advances have opened new possibilities for improving muscle assessment and nutritional monitoring. In recent years, artificial intelligence (AI), image processing techniques, and wearable smart devices have emerged as innovative tools for assessing muscle mass and monitoring malnutrition. In particular, deep learning–based image processing algorithms can automatically measure muscle CSA and MT from ultrasound images, reducing observer-dependent variability and improving reliability [17]. Similarly, automated ultrasound image segmentation tools such as DeepACSA (focused on lower limb muscles) and U-Net–based models have demonstrated high accuracy (Dice scores approximately 90 %) in delineating muscle boundaries in musculoskeletal ultrasound images, including studies on forearm muscles. Although these examples focus on the upper limb, similar deep learning–based segmentation approaches have been successfully applied to lower limb muscles such as the RF, demonstrating comparable accuracy rates [18,19]. These AI-based methods, when integrated into portable ultrasound devices, offer potential for early and objective detection of muscle wasting, especially in palliative care settings where bedside imaging is essential. Moreover, wearable biosensors that continuously monitor physical activity and energy expenditure complement ultrasound findings, providing a holistic approach to evaluating nutrition–muscle interactions.

In our study, the mean age of participants was 74.09 years, the mean BMI was 22.39 kg/m^2^, and the mean NRS-2002 score was 5.01. These findings indicate that the study population consisted of elderly, underweight individuals at high risk of advanced malnutrition. Among patients who achieved their caloric targets, the mean time to goal attainment was 7 days, suggesting that effective nutritional monitoring is feasible.

Achieving nutritional goals is often challenging in patients with terminal cancer. This population frequently experiences nutritional difficulties due to multifactorial causes such as anorexia–cachexia syndrome, gastrointestinal obstruction, chemotherapy-induced nausea and vomiting, and pain. In addition, factors such as vascular access issues, the need to avoid hyperalimentation, the risk of metabolic complications (e.g., hyperglycemia, electrolyte imbalances), and treatment limitations arising from the patient or medical team can hinder the effective implementation of nutritional support [20–22]. This was also reflected in our study, as 25.26% of patients failed to achieve their targeted caloric intake. This group is considered to be at higher risk of muscle mass loss. Even short-term energy deficits may accelerate muscle wasting in elderly individuals and in those with advanced-stage disease [23].

In our cohort, lung cancer was the most frequently observed malignancy, consistent with previous palliative care studies in which its high incidence and advanced stage at diagnosis make it the predominant cancer type [24,25]. The distribution of other malignancies similarly reflected patterns seen in terminal-stage populations, where aggressive cancers such as those of the central nervous system, stomach, and pancreas are frequently represented. Most patients in our study had performance scores indicating substantial functional limitations, ranging from difficulty in walking to being bedridden. Since only terminal-stage patients whose systemic anticancer treatments had been discontinued were admitted to our palliative care unit, the predominance of low performance status was expected. Such reduced mobility not only reflects the severity of the underlying disease but also contributes to accelerated muscle wasting and nutritional decline [26]. This interplay among advanced disease, impaired functional capacity, and sarcopenia underscores the multidimensional challenges inherent in managing late-stage oncology patients.

Muscle wasting is an almost inevitable process in patients with terminal-stage cancer. In our study, a noticeable reduction in muscle measurements was observed across participants, regardless of whether they achieved their nutritional targets. This finding highlights the detrimental impact of catabolic processes and inflammatory responses in the terminal stage, which may lead to muscle loss despite nutritional support. In the study by Rollinson et al., ICU patients were categorized into two groups: critically ill and relatively stable. The RF muscle was assessed using ultrasound on days 1, 4, and 7. The study found that the critically ill group exhibited significantly greater muscle degradation [27]. Similarly, Garcia-Grimaldo et al. aimed to quantify skeletal muscle loss and identify its risk factors in ICU patients hospitalized with COVID-19. Using two abdominal CT scans performed 10 days apart at the lumbar-3 (L3) level, they measured CSA and detected an 11% loss in muscle mass within 10 days [28]. We were unable to identify studies in the literature that specifically examined a comparable patient population receiving palliative care. Although the patients in our study were not in the intensive care unit (ICU) but were instead receiving terminal-stage palliative care, both groups shared common clinical features such as systemic inflammation, limited mobility, and nutritional deficiencies. Therefore, progressive muscle loss can be expected in both populations.

In our study, comparisons of muscle indices between participants who achieved and those who did not achieve their caloric targets showed significantly greater muscle loss across all parameters, particularly CSA-I, in the group that failed to meet caloric goals. These findings suggest that muscle loss is closely related to energy intake and that failure to achieve caloric targets poses a serious risk to maintaining muscle integrity. In a multicenter study by Martin et al. involving patients with advanced gastrointestinal cancer, the incidence of sarcopenia and treatment-related toxicities was higher among those with low caloric and protein intake [29]. Similarly, Mourtzakis et al. reported that energy deficits are directly associated with severe muscle loss, which may lead to irreversible physical deterioration, particularly in the later stages of the disease [30]. Our findings, consistent with existing literature, demonstrate that insufficient caloric intake leads not only to reductions in body weight but also to a rapid decline in lean and functional muscle mass. Therefore, even in terminal-stage patients, achieving energy targets as early and adequately as possible is critically important not only for prolonging survival but also for preserving quality of life and functional capacity [31,32].

In our study, a significant decrease in RF-CSA-I was observed among terminal-stage cancer patients who failed to achieve their caloric targets. This finding aligns with current evidence indicating that inadequate energy intake suppresses muscle protein synthesis and accelerates muscle loss. The RF muscle is described in the literature as one of the most sensitive muscles to changes in nutritional status. In particular, studies involving ICU and cancer populations have reported strong correlations of RF-MT and RF-CSA with energy balance [33]. For instance, in a prospective study by Tillquist et al., a rapid reduction in RF-MT was observed in patients with energy deficiency, which was associated with functional status [15]. Similarly, Prado et al. reported a significant association between low energy intake and RF-CSA loss in patients with advanced gastrointestinal cancer [34]. Additionally, RF-CSA measured via ultrasound has been shown to strongly correlate with CT findings, reinforcing its value as a reliable, noninvasive biomarker for monitoring muscle mass [35].

In contrast, the parameters related to the BB muscle exhibited a weaker but still significant relationship with caloric intake. This difference may be attributed to physiological variations in functional load and usage intensity between upper and lower extremity muscles. As a major lower limb muscle, the RF plays a key role in postural balance and locomotion, making it more susceptible to systemic catabolic stress and inadequate protein intake, and therefore more likely to exhibit early and pronounced losses [36].

A recent study by Catikkas and Binay Safer suggested that the BB muscle may serve as a prognostic indicator, particularly in sarcopenic individuals [37]. The same study also reported that the RF muscle is rich in type II muscle fibers. Type II fibers generate high power and rapid contractions over short periods and rely heavily on glycogen as an energy source. Consequently, in states of energy deficiency, the body rapidly breaks down these fibers. This catabolic process becomes more pronounced under inadequate protein intake, leading to accelerated atrophy [10].

Fearon K et al. emphasized that cancer cachexia is not solely the result of caloric deficiency but is also driven by increased protein catabolism caused by systemic inflammation. Therefore, adequate protein intake is as critical as energy intake in this context [38]. The literature, particularly the ESPEN and PROT-AGE guidelines, underscores the importance of maintaining a daily protein intake of ≥1.5 g/kg in patients with cancer to reduce muscle wasting and prevent functional decline [12,39]. According to the ESPEN guidelines for older adults, a daily protein intake of ≥1.5 g/kg is recommended to maximize muscle protein synthesis, whereas lower intakes fail to achieve this effect [40].

In our study, among patients who achieved their caloric targets, those receiving ≥1.5 g/kg/day of protein demonstrated lower muscle loss in both RF and BB muscles. Muscle loss in the RF was significantly attenuated, as reflected by both CSA and MT indices, and this association was further supported by moderate positive correlations. BB-CSA-I also showed significant benefits of higher protein intake, whereas BB-MT-I was less affected. In contrast, no significant differences in subcutaneous fat tissue thickness (SFTT) were observed in either the RF or BB regions according to protein intake level. Taken together, these findings suggest that although lower-extremity muscles appear more sensitive to protein deficiency and metabolic stress, both upper- and lower-extremity muscles benefit from adequate protein intake, highlighting the importance of achieving ≥1.5 g/kg/day to attenuate muscle loss.

To explore this relationship beyond a predefined threshold, we conducted correlation analyses between protein intake and muscle indices. The results provided a complementary perspective to categorical comparisons: while group-based analysis using the ≥1.5 g/kg/day threshold identified clear differences in muscle preservation, evaluating protein intake as a continuous variable revealed a graded relationship—moderate positive associations with RF indices and weaker but directionally similar trends for BB indices. These findings, consistent with the literature, indicate that the beneficial effect of high protein intake on muscle preservation is not confined to a specific threshold but increases progressively with intake, further emphasizing the clinical importance of optimizing protein provision in terminal-stage cancer patients [41]. SFTT-I values in the RF and BB regions did not show statistically significant variation according to protein intake levels. This finding suggests that protein intake directly affects muscle tissue, whereas subcutaneous fat tissue is more likely to change in response to overall energy balance [42].

Based on the analysis, muscle loss rates did not differ significantly regardless of the feeding method used. The literature suggests that oral and enteral nutrition may help preserve gastrointestinal integrity, reduce inflammation, and lessen muscle loss in patients [43]. Therefore, at the outset of our study, we hypothesized that patients receiving parenteral nutrition would exhibit more pronounced muscle loss. Greater muscle loss was observed in all RF muscle index measurements and in BB-CSA-I among patients receiving parenteral nutrition compared with other feeding routes; however, this difference did not reach statistical significance. This finding may be attributed to the limited sample size and the unequal distribution between groups.

In addition to these analyses, a multivariable linear regression model was conducted to evaluate the combined effects of age, sex, NRS-2002 score, feeding route, and ECOG performance status on changes in the RF muscle area index. This model did not reach statistical significance (F = 1.368; p = 0.215; adjusted R^2^ = 0.034). The Durbin–Watson coefficient was 1.870, close to the ideal value of 2, indicating no autocorrelation in the residuals and supporting the reliability of the regression model. These findings suggest that demographic and clinical factors such as age, sex, nutritional risk, feeding route, and functional status did not independently predict changes in muscle mass in our cohort, which may also reflect the limited sample size and statistical power. The absence of significant associations indicates that the influence of nutritional adequacy on RF muscle preservation is not confounded by these variables, reinforcing the conclusion that achieving caloric and protein targets plays a more decisive role in maintaining muscle integrity than baseline demographic characteristics or feeding routes. This finding highlights the importance of tailored nutritional interventions in preserving muscle mass.

The negative correlation observed between muscle loss and the number of days required to reach maximum energy targets in the group that achieved adequate caloric intake is noteworthy. This finding suggests that not only total caloric intake but also the timeliness of achieving adequate nutrition is a critical factor in muscle preservation. Delayed initiation or suboptimal progression of nutritional support may result in irreversible muscle loss in terminal-stage patients [30,44].

In conclusion, this study demonstrated that early achievement of caloric targets—particularly adequate protein intake—plays a decisive role in preserving muscle mass in terminal-stage cancer patients. Muscle US is gaining importance in clinical practice as a reliable, practical, and noninvasive tool for monitoring this process. Our findings suggest that the RF muscle is a more sensitive indicator of energy deficiency, supporting the prioritization of lower-extremity muscle assessment in monitoring palliative care patients.

Previous studies have examined muscle US in various clinical settings; however, none have specifically investigated the relationship between achieving individualized energy and protein targets and changes in muscle mass in terminal-stage palliative care patients. For example, Kaya et al. evaluated RF-CSA and RF-MT in palliative care patients to predict 3-month mortality, without assessing nutritional intake [7]. Fernández-Jiménez et al. assessed malnutrition or sarcopenia using ultrasound in cancer patients, but their studies were not conducted in terminal-stage palliative settings and did not analyze individualized macronutrient goals [45]. Likewise, Tume et al. investigated the association between nutrition and ultrasound-measured muscle changes in pediatric intensive care patients, representing a fundamentally different population [46]. Therefore, to our knowledge, the present study is the first to comprehensively evaluate the impact of achieving individualized caloric and protein goals on ultrasound-based muscle measurements specifically in terminal-stage palliative care patients. Our findings indicate that a protein intake of ≥1.5 g/kg/day plays a critical role in preserving muscle integrity, with the RF muscle showing more pronounced preservation. While this relationship has been more commonly studied in intensive care or surgical patient groups [15,34], our study is the first to examine it in detail in terminal-stage palliative care patients. Therefore, our study provides strong evidence for the importance of early and adequate protein–energy support in this population, objectively demonstrated through ultrasound measurements. In this respect, the study offers a unique contribution both to the palliative care literature and to the clinical application of ultrasound-based muscle monitoring methods.

## 5. Limitations

This study has several limitations, primarily related to its retrospective design. First, although changes in muscle mass were evaluated in relation to nutritional status, other important factors influencing muscle preservation—such as mobilization level, physical activity, and exercise interventions—were not recorded. The absence of these data limits the ability to isolate the effect of nutrition from other determinants of muscle loss. Second, the retrospective design restricted control over potential confounders and led to occasional gaps in clinical data.

Another limitation is the relatively short follow-up period, with only two muscle ultrasound assessments conducted over approximately 10 days. This timeframe allowed detection of acute changes but may not fully capture the longer-term trajectory of muscle wasting in terminal-stage cancer patients. Additionally, the modest sample size, coupled with unequal distribution across feeding route subgroups, may have limited the statistical power to detect certain associations. Although an a priori power analysis indicated that the overall sample size was adequate, certain critical subgroup comparisons remained underpowered. Therefore, some nonsignificant findings may partly reflect a type II error rather than a true absence of effect.

Despite these limitations, the methodological strength of having all ultrasound measurements performed by the same physician with the same device ensured consistency and reduced interobserver variability. Future studies should adopt a prospective, multicenter design with larger sample sizes, extended follow-up periods, and comprehensive assessments of mobility and activity levels. Integrating objective activity monitoring (e.g., wearable sensors) and biochemical markers of inflammation could provide a more detailed understanding of the multifactorial drivers of muscle loss.

Finally, excluding patients who died before the second measurement may have introduced survival bias, potentially leading to an overrepresentation of individuals with relatively better short-term prognoses. As a result, the observed associations between nutritional intake and muscle mass changes may not fully reflect the patterns in the most critically ill subset of terminal-stage patients.

## 6. Conclusion

This study demonstrated that adequate energy intake—particularly high-protein intake—significantly contributes to reducing muscle loss in terminal-stage cancer patients. US, particularly for assessing the RF muscle, proved to be a reliable, practical, and reproducible tool for muscle assessment.

Our findings highlight that nutritional therapy should not be neglected, even in irreversible phases such as the terminal stage, and that achieving targeted protein and caloric levels early is essential.

Personalizing nutritional strategies in palliative care is a fundamental approach that may influence not only survival but also patients’ functional status and dependency levels.

## Figures and Tables

**Figure:**
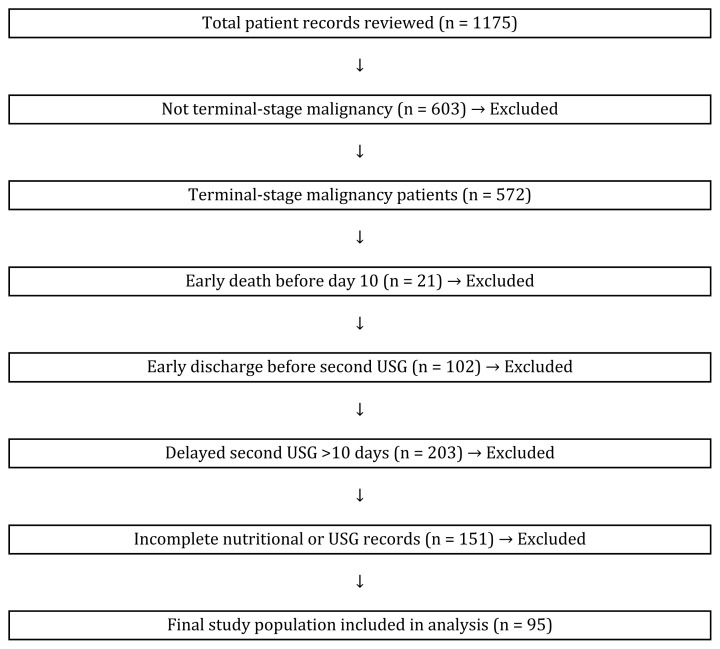
Patient selection process.

**Table 1:** Descriptive statistics of participants’ demographic, clinical, and muscle measurement variables.

Variable	Category	n/Mean± SD	%/Median (Min–Max)
Sex	Male	53	55.79
	Female	42	44.21
Achievement of Caloric Target	Yes	71	74.74
	No	24	25.26
Nutrition Route	PN	23	24.21
	EN	29	30.52
	OR	33	34.73
	PN+OR	10	10.52
Type of Cancer	Lung Cancer	22	23.1
	CNS	12	12.6
	Gastric Cancer	10	10.5
	Pancreatic Cancer	8	8.4
	Prostate Cancer	8	8.4
	Breast Cancer	6	6.3
	Endometrial Cancer	5	5.3
	Colon Cancer	5	5.3
	HCC	5	5.3
	Nasopharyngeal Cancer	3	3.2
	Malignant melanoma	2	2.2
	Bladder Cancer	2	2.1
	Overian Cancer	2	2.1
	Fibrosarcoma	1	1.1
	Cholangiocarcinoma	1	1.1
	Leimyosarcoma	1	1.1
	Rectal Cancer	1	1.1
	Cervical Cancer	1	1.1
ECOG performance status	0	7	7.4
	1	7	7.4
	2	28	29.4
	3	29	30.5
	4	24	25.2
Age (Mean/SD)		74.09 ± 10.365	74.00 (48–96)
NRS-2002 (Mean/SD)		5.01 ± 0.893	5.00 (3–6)
Days to Reach Caloric Target (Mean/SD)		7.00 ± 2.806	8.00 (1–10)
BMI (Mean/SD)		22.39 ± 5.079	22.00 (15–48)
1. RF-SFTT-I		0.318 ± 0.212	0.249 (0.078–0.888)
2. RF-SFTT-I		0.282 ± 0.187	0.201 (0.053–0.865)
1. RF-MT-I		0.262 ± 0.101	0.248 (0.103–0.618)
2. RF-MT-I		0.232 ± 0.091	0.223 (0.083–0.631)
1. RF-CSA-I		0.749 ± 0.216	0.723 (0.270–1.706)
2. RF-CSA-I		0.682 ± 0.196	0.680 (0.239–1.242)
1. BB-SFTT-I		0.116 ± 0.089	0.085 (0.036–0.457)
2. BB-SFTT-I		0.110 ± 0.086	0.081 (0.033–0.446)
1. BB-MT-I		0.518 ± 0.114	0.523 (0.288–0.875)
2. BB-MT-I		0.478 ± 0.109	0.471 (0.254–0.723)
1.BB-CSA-I		1.493 ± 0.368	1.451 (0.696–2.770)
2. BB-CSA-I		1.384 ± 0.367	1.375 (0.616–2.410)

PN: parenteral nutrition; EN: enteral nutrition; OR: oral nutrition; CNS: central nervous system; HCC: hepatocellular carcinoma; ECOG: Eastern Cooperative Oncology Group; NRS-2002: Nutrition Risk Screening 2002; BB-CSA-I: cross-sectional area index of the biceps brachii muscle; BB-MT-I: muscle thickness index of the biceps brachii muscle; BB-SFTT-I: subcutaneous fat tissue thickness index of the biceps brachii muscle; RF-CSA-I: cross-sectional area index of the rectus femoris muscle; RF-MT-I: muscle thickness index of the rectus femoris muscle; RF-SFTT-I: subcutaneous fat tissue thickness index of the rectus femoris muscle.

n: number of observations; M: mean; SD: standard deviation; Med: median; Min: minimum; Max: maximum.

Note: Categorical variables are presented as n (%) and continuous variables are expressed as mean ± SD or median (Min–Max).

**Table 2:** Comparison of muscle index differences between participants who achieved and those who did not achieve caloric targets.

Variable	Achieved (n = 71)		Not Achieved (n = 24)		Test Statistic	p
	Mean ± SD	Med (Q1–Q3)	Mean ± SD	Med (Q1–Q3)		
BB-CSA-I (difference)	−0.054 ± 0.138	−0.050 (−0.105 – 0.010)	−0.272 ± 0.145	−0.226 (−0.364 – −0.159)	6.619	<0.001^t^
BB-MT-I (difference)	−0.024 ± 0.045	−0.013 (−0.044 –0.002)	−0.089 ± 0.082	−0.062 (−0.121 – −0.030)	3.725	0.001^t^
BB-SFTT-I (difference)	−0.002 ± 0.027	−0.002 (−0.014 – 0.009)	−0.018 ± 0.022	−0.014 (−0.033 – −0.001)	−2.694	0.007^u^
RF-CSA-I (difference)	−0.041 ± 0.082	−0.033 (−0.075 – −0.007)	−0.141 ± 0.125	−0.112 (−0.195 – −0.045)	3.626	0.001^t^
RF MT-I (difference)	−0.017 ± 0.038	−0.014 (−0.037 –0.012)	−0.067 ± 0.057	−0.052 (−0.104 – −0.021)	4.028	<0.001^t^
RF-SFTT-I (difference)	−0.021 ± 0.067	−0.018 (−0.042 – 0.016)	−0.079 ± 0.086	−0.065 (−0.102 – −0.025)	−3.573	<0.001^u^

BB-CSA-I: cross-sectional area index of the biceps brachii muscle; BB-MT-I: muscle thickness index of the biceps brachii muscle; BB-SFTT-I: subcutaneous fat tissue thickness index of the biceps brachii muscle; RF-CSA-I: cross-sectional area index of the rectus femoris muscle; RF-MT-I: muscle thickness index of the rectus femoris muscle; RF-SFTT-I: subcutaneous fat tissue thickness index of the rectus femoris muscle.

M: mean; SD: standard deviation; Med: median; Q1: 25th percentile; Q3: 75th percentile; t: independent-sample t test; u: Mann–Whitney U test.

**Table 3:** Comparison of muscle index differences according to daily protein intake among participants who achieved caloric targets.

Variable	<1.5 g/kg/day (n = 35)		≥1.5 g/kg/day (n = 36)		Test statistic	p
	Mean ± SD	Med (Q1–Q3)	Mean ± SD	Med (Q1–Q3)		
BB-CSA-I (difference)	−0.087 ± 0.137	−0.059 (−0.172 – −0.025)	−0.023 ± 0.132	−0.039 (−0.074 – 0.044)	−2.010	0.048^t^
BB-MT-I (difference)	−0.035 ± 0.045	−0.027 (−0.059 – −0.007)	−0.012 ± 0.043	−0.005 (−0.027 – 0.013)	2.663	0.008^u^
BB-SFTT-I (difference)	0.005 ± 0.027	0.002 (−0.011 – 0.015)	−0.008 ± 0.025	−0.003 (−0.018 – 0.004)	−1.737	0.082^u^
RF-CSA-I (difference)	−0.083 ± 0.069	−0.066 (−0.131 – −0.032)	−0.001 ± 0.073	−0.008 (−0.049 – 0.020)	−4.880	<0.001^t^
RF-MT-I (difference)	−0.034 ± 0.041	−0.029 (−0.063 – 0.004)	−0.002 ± 0.028	−0.001 (−0.021 – 0.015)	−3.867	<0.001^t^
RF-SFTT-I (difference)	−0.035 ± 0.081	−0.022 (−0.062 – 0.016)	−0.008 ± 0.046	−0.017 (−0.038 – 0.019)	1.001	0.317^u^

BB-CSA-I: cross-sectional area index of the biceps brachii muscle; BB-MT-I: muscle thickness index of the biceps brachii muscle; BB-SFTT-I: subcutaneous fat tissue thickness index of the biceps brachii muscle; RF-CSA-I: cross-sectional area index of the rectus femoris muscle; RF-MT-I: muscle thickness index of the rectus femoris muscle; RF-SFTT-I: subcutaneous fat tissue thickness index of the rectus femoris muscle.

M: mean; SD: standard deviation; Med: median; Q1: 25th percentile; Q3: 75th percentile; t: independent-sample t test; u: Mann–Whitney U test.

**Table 4:** Correlations between daily protein intake and muscle index differences among participants who achieved their caloric target.

Variable	Value	Protein intake (g/kg)
BB-CSA-I (difference)	r	0.202
	p	0.092
BB-MT-I (difference)	r	0.193
	p	0.107
BB-SFTT-I (difference)	r	−0.139
	p	0.247
RF-CSA-I (difference)	r	0.507
	p	<0.001
RF-MT-I (difference)	r	0.485
	p	<0.001
RF-SFTT-I (difference)	ρ	0.249
	p	0.036

r: Pearson correlation coefficient; ρ: Spearman’s correlation coefficient.

**Table 5:** Comparison of muscle index differences according to participants’ feeding routes.

Variable	PN (n = 23)		EN (n = 29)		OR (n = 33)		PN+ OR (n = 10)		Test statistic	p
	Ort ± SS	Med (Q1–Q3)	Mean ± SD	Med (Q1–Q3)	Mean ± SD	Med (Q1–Q3)	Mean ± SD	Med (Q1–Q3)		
BB-CSA-I (difference)	−0.106 ± 0.177	−0.072 (−0.220 – 0.004)	−0.110 ± 0.159	−0.066 (−0.249 – −0.008)	−0.092 ± 0.184	−0.055 (−0.164 – −0.020)	−0.173 ± 0.122	−0.169 (−0.222 – −0.088)	0.590	0.623^F^
BB-MT-I (difference)	−0.023 ± 0.034	−0.021 (−0.039 – 0.000)	−0.037 ± 0.050	−0.029 (−0.055 – −0.007)	−0.051 ± 0.082	−0.032 (−0.090 – 0.001)	−0.051 ± 0.073	−0.036 (−0.076 – −0.009)	1.366	0.713^KW^
BB-SFTT-I (difference)	0.002 ± 0.031	0.000 (−0.026 – 0.014)	−0.006 ± 0.022	−0.004 (−0.019 – 0.007)	−0.006 ± 0.022	−0.003 (−0.014 – 0.005)	−0.023 ± 0.035	−0.015 (−0.041 – 0.005)	2.094	0.107^F^
RF-CSA-I (difference)	−0.055 ± 0.071	−0.037 (−0.082 – 0.007)	−0.062 ± 0.090	−0.052 (−0.108 – 0.013)	−0.052 ± 0.097	−0.043 (−0.114 – −0.013)	−0.152 ± 0.178	−0.077 (−0.242 – −0.027)	2.874	0.411^KW^
RF-MT-I (difference)	−0.033 ± 0.047	−0.022 (−0.047 – 0.001)	−0.019 ± 0.039	−0.015 (−0.033 – 0.014)	−0.028 ± 0.046	−0.029 (−0.066 – 0.010)	−0.061 ± 0.074	−0.025 (−0.099 – 0.015)	1.195	0.133^F^
RF-SFTT-I (difference)	−0.021 ± 0.049	−0.022 (−0.054 – 0.008)	−0.030 ± 0.086	−0.022 (−0.058 – 0.019)	−0.035 ± 0.061	−0.024 (−0.081 – 0.004)	−0.090 ± 0.117	−0.056 (−0.138 – 0.024)	4.583	0.205^KW^

PN: parenteral nutrition; EN: enteral nutrition, OR: oral nutrition; BB-CSA-I: cross-sectional area index of the biceps brachii muscle; BB-MT-I: muscle thickness index of the biceps brachii muscle; BB-SFTT-I: subcutaneous fat tissue thickness index of the biceps brachii muscle; RF-CSA-I: cross-sectional area index of the rectus femoris muscle; RF-MT-I: muscle thickness index of the rectus femoris muscle; RF-SFTT-I: subcutaneous fat tissue thickness index of the rectus femoris muscle.

M: mean; SD: standard deviation; Med: median; Q1: 25th percentile; Q3: 75th percentile.

KW: Kruskal–Wallis test; F: one-way ANOVA test.

**Table 6:** Multivariable linear regression analysis of RF-CSA-I differences.

Variable	B	SE	Std (B)	t	p	CI (B)		VIF
						Lower bound	Lower bound	
Constant	−0.005	0.092		−0.050	0.960	−0.188	0.179	
Age	0.000	0.001	−0.013	−0.099	0.922	−0.003	0.002	1.603
Sex (ref=Male) Sex	−0.016	0.022	−0.078	−0.744	0.459	−0.060	0.027	1.079
NRS-2002	−0.003	0.015	−0.029	−0.234	0.815	−0.032	0.026	1.538
Feeding Route (ref: PN)								
Feeding Route (dummy EN)	−0.022	0.031	−0.097	−0.700	0.486	−0.083	0.040	1.861
Feeding Route (dummy OR)	−0.001	0.032	−0.004	−0.027	0.978	−0.064	0.062	2.066
Feeding Route (dummy PN+OR)	−0.081	0.044	−0.242	−1.853	0.067	−0.169	0.006	1.659
ECOG performance status	−0.001	0.010	−0.008	−0.073	0.942	−0.020	0.018	1.131
DW = 1.870, F= 1.368, p = 0.215, Adj R^2^ = 0.034								

NRS-2002: Nutrition Risk Screening 2002; PN: parenteral nutrition; EN: enteral nutrition; OR: oral nutrition; ECOG: Eastern Cooperative Oncology Group; B: regression coefficient; SE: standard error; Std (B): standardized beta coefficient; t: t-test statistic; CI (B): 95% confidence interval for B coefficients; VIF: variance inflation factor; DW: Durbin–Watson coefficient; Adj. R^2^: adjusted R^2^.

**Table 7:** Correlations between days to reach maximum caloric intake and muscle index differences in the group that achieved caloric targets.

Variable	Value	Number of days to reach maximum caloric intake
BB-CSA-I	r	−0.419
	p	<0.001
BB-MT-I	ρ	−0.468
	p	<0.001
BB-SFTT-I	r	−0.137
	p	0.185
RF-CSA-I	ρ	−0.273
	p	0.007
RF-MT-I	r	−0.381
	p	<0.001
RF-SFTT-I	ρ	−0.177
	p	0.086

BB-CSA-I: cross-sectional area index of the biceps brachii muscle; BB-MT-I: muscle thickness index of the biceps brachii muscle; BB-SFTT-I: subcutaneous fat tissue thickness index of the biceps brachii muscle; RF-CSA-I: cross-sectional area index of the rectus femoris muscle; RF-MT-I: muscle thickness index of the rectus femoris muscle; RF-SFTT-I: subcutaneous fat tissue thickness index of the rectus femoris muscle.

r: Pearson correlation coefficient; ρ: Spearman’s correlation coefficient.

## Data Availability

The datasets generated and analyzed during the current study are included in this article. Data associated with this study are available from the corresponding authors upon request.
